# Supplementary material for: Intestinal IL-22RA1 signaling regulates intrinsic and systemic lipid and glucose metabolism to alleviate obesity-associated disorders
Source: Nat Commun. 2024 Feb 21;15:1597. doi: 10.1038/s41467-024-45568-6 (PMC10881576; doi:10.1038/s41467-024-45568-6)
Supplement: Supplementary file 1 — Supplementary Information [file 41467_2024_45568_MOESM1_ESM.pdf]

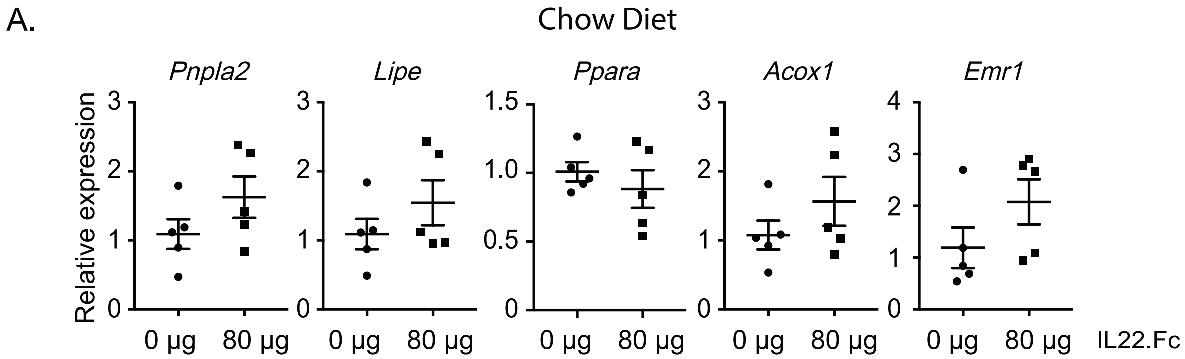

Supplementary Fig. 1. RT-PCR analysis of metabolic and inflammatory genes from epididymal WAT of IL-22.Fc injected mice. S1 represents 2 independent experiments and N = 5 cre- and 5 cre+ mice. Data are presented as mean ± SEM in all graphs. Mann-Whitney test, two-tailed.

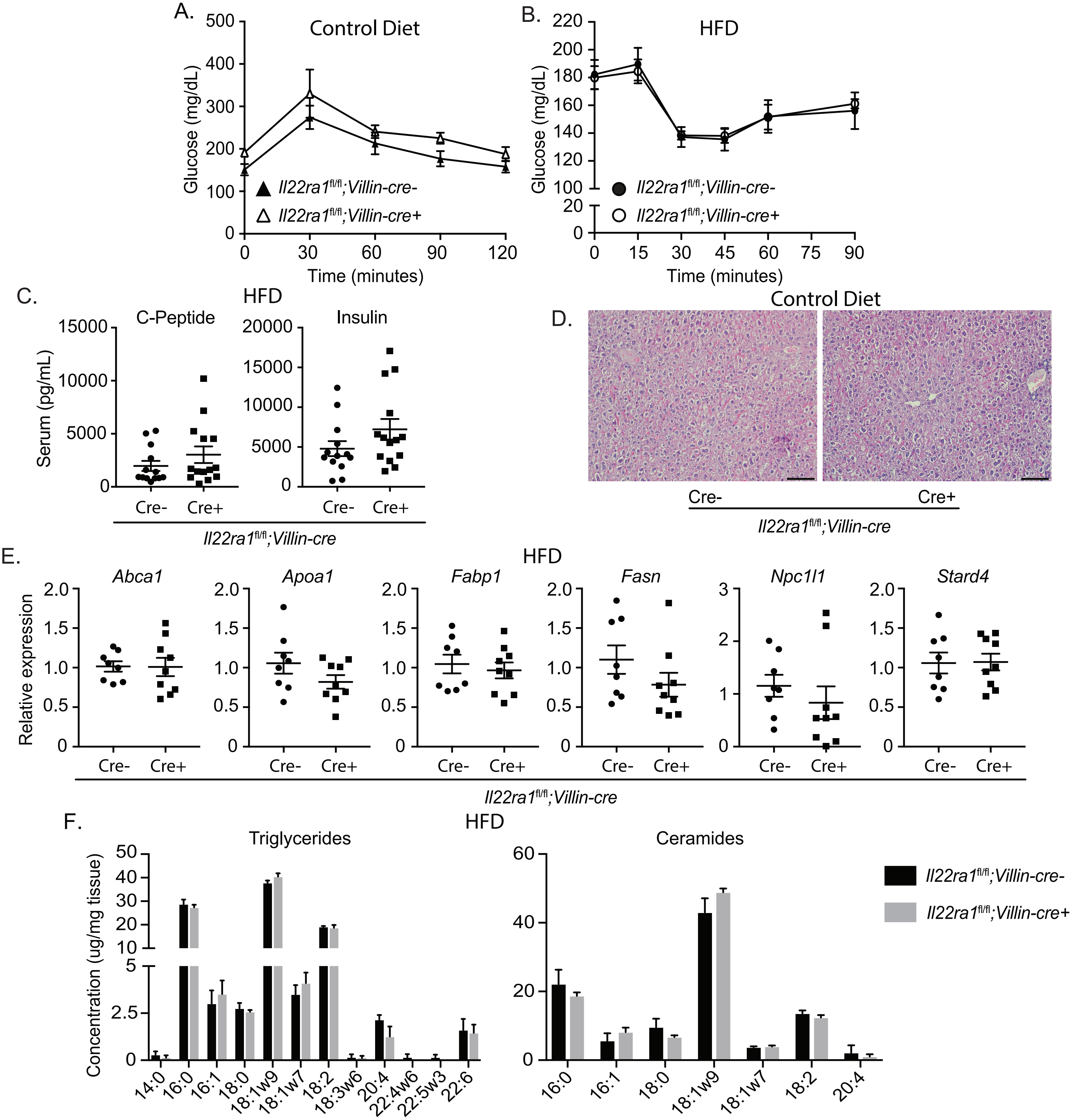

Supplementary Fig. 2. Intestinal IL-22RA1-mediated regulation of hepatic function. (A) GTT was conducted in 16 week control diet fed Il22ra1fl/fl;Villin-cre mice. (B) Insulin tolerance test from Il22ra1fl/fl;Villin-cre mice. (C) Luminex multiplex analysis of c-peptide and insulin from serum isolated from Il22ra1fl/fl;Villin-cre mice. (D) H&E staining of liver from control diet fed Il22ra1fl/fl;Villin-cre mice mice. (E) RT-PCR analysis of metabolic genes from the liver of HFD fed Il22ra1fl/fl;Villin-cre mice. (F) Individual concentration of triglycerides and ceramides from liver tissues of HFD fed Il22ra1fl/fl;Villin-cre mice.

S2A, D are representative of 2 independent experiments. S2A represents N = 6 cre- and 4 cre+ mice. S2D represents 5 cre- and 4 cre+ mice. S2B represents 2 independent experiments and N = 6 cre- and 6 cre+ mice. S2C represents 5 independent experiments and N = 13 cre- and 14 cre+ mice. S2E represents 3 independent experiments and N = 8 cre- and 9 cre+ mice. S2F represents 3 independent experiments and N = 4 cre- and 5 cre+ mice. 2-way ANOVA with Sidak multiple comparison in A-B, F; Mann-Whitney test, two-tailed in C, F. Scale bar = 50 μm. Data are presented as mean ± SEM in all graphs.

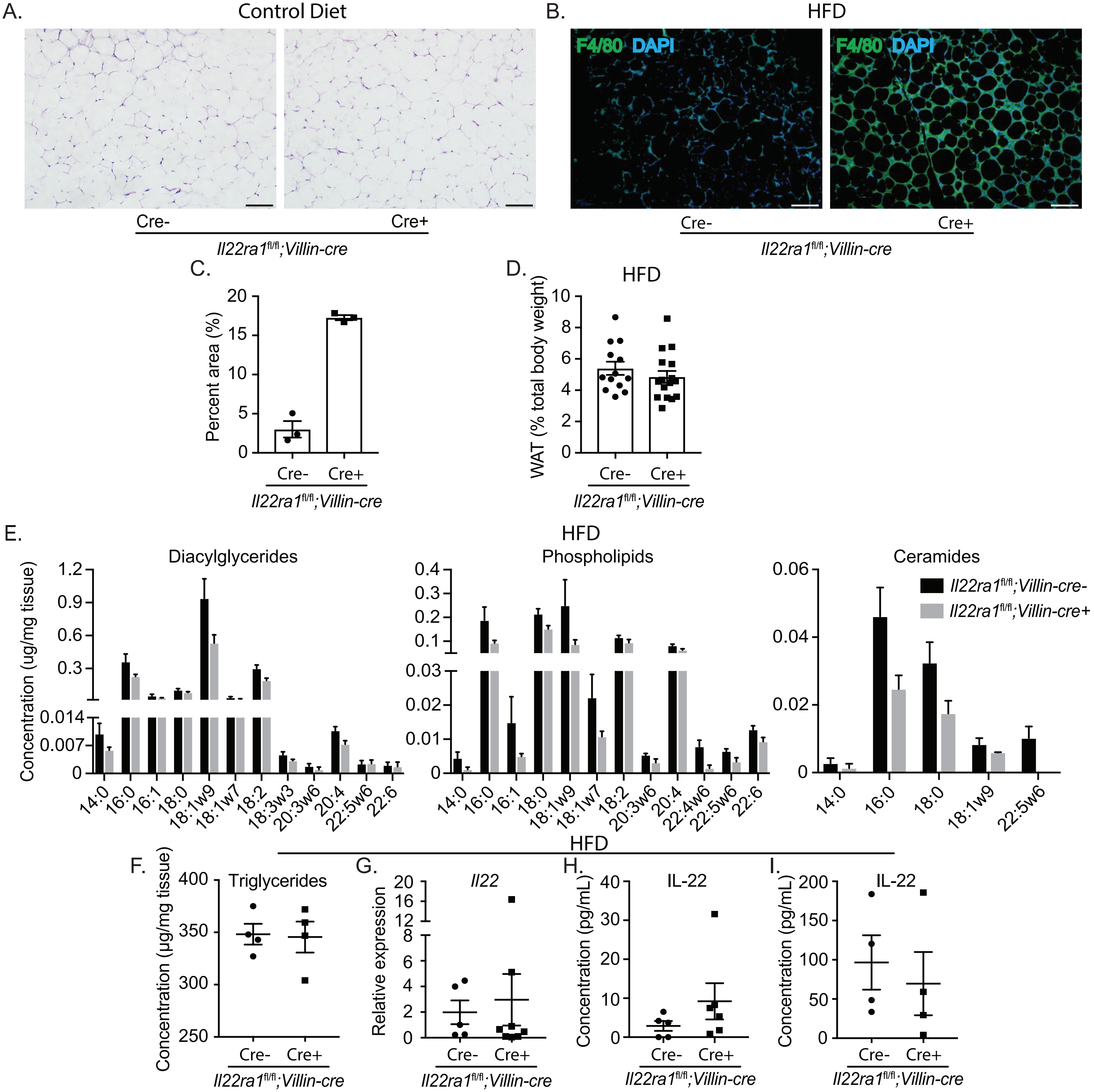

Supplementary Fig. 3. Intestinal IL-22RA1-mediated regulation of WAT and systemic functions. (A) H&E staining of epididymal WAT from control diet fed Il22ra1fl/fl;Villin-cre mice. (B) Immunofluorescence analysis of epididymal WAT macrophage accumulation from HFD fed mice. (C) Quantification of F4/80 staining in figure 3B. (D) Epididymal WAT fat pad weight. (E) Individual concentrations of diacylglycerides, phospholipids, and ceramides from epydidymal WAT of HFD fed Il22ra1fl/fl;Villin-cre mice. (F) Gas chromotography analysis of epididymal WAT triglyceride levels. (G) RT-PCR of ileal Il22 expression. (H) ELISA of hepatic IL-22. (I) Luminex multiplex analysis of serum IL-22.

S3A, E-I represent 2 independent experiments. S3A represents N = 6 cre- and 4 cre+ mice. S3B – C represent N = 3 cre- and 3 cre+ mice from 3 independent experiments. S3D represents N = 13 cre- and 16 cre+ mice from 7 independent experiments. S3E is generated from 4 cre- and 4 cre+ mice. S3F, I represent N = 4 cre- and 4 cre+ mice. S3G represents 5 cre- and 8 cre+ mice. S3H represents N = 5 cre- and 6 cre+ mice. Data are presented as mean ± SEM in all graphs. 2-way ANOVA in SF; Mann Whitney test, two-tailed in S3C-D, F-I. Scale bar (S3A) = 50 µm, (S3B) = 100 µm.

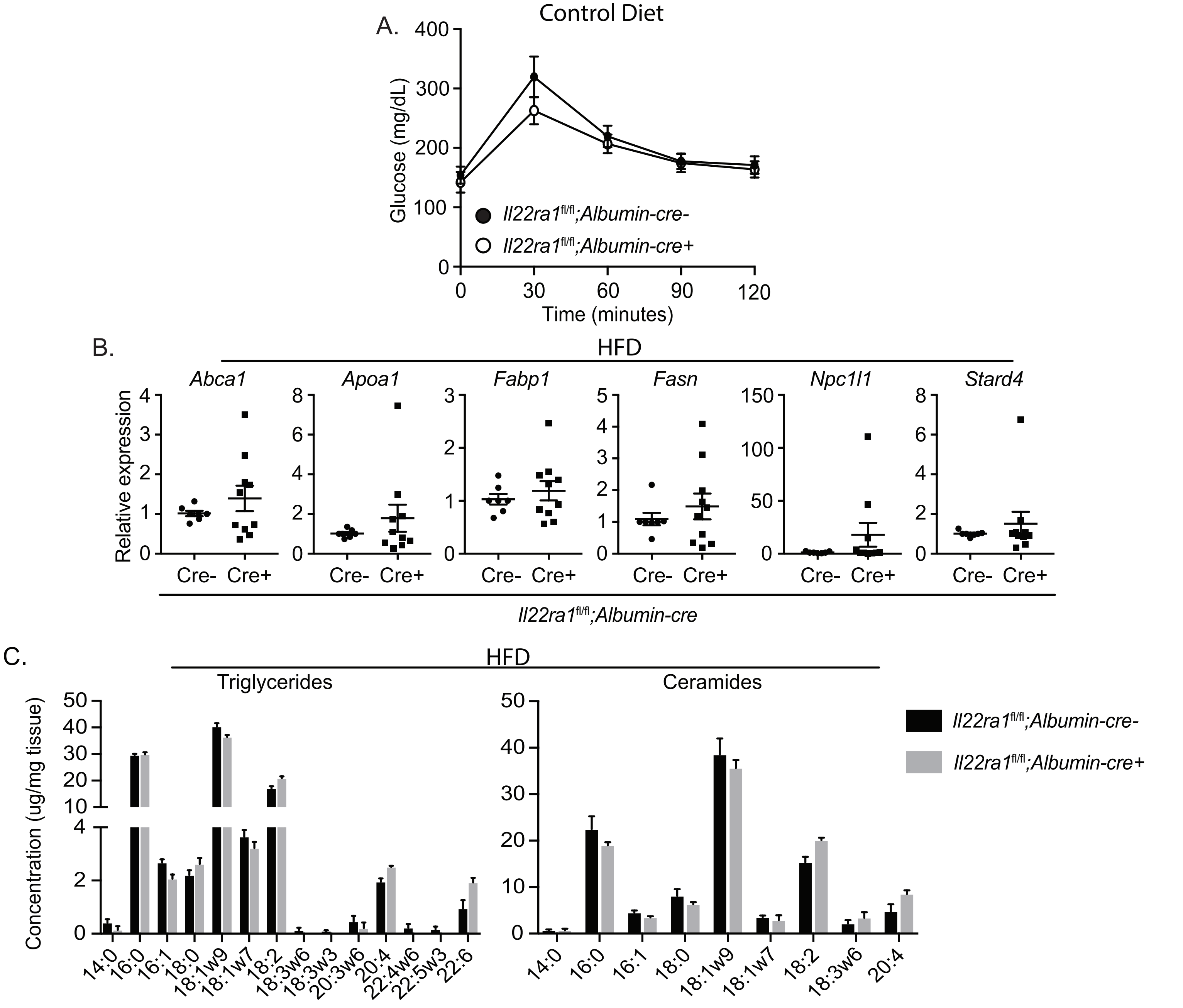

Supplementary Fig. 4. Systemic and hepatic effects of liver-specific IL-22RA1 signaling. (A) GTT was conducted in 16 week control diet fed Il22ra1fl/fl;Albumin-cre mice (N = 9 cre- and 8 cre+) mice. (B) RT-PCR analysis of metabolic genes from the liver of Il22ra1fl/fl;Albumin-cre mice fed 16 weeks HFD. (C) Individual concentrations of triglycerides and ceramides from the liver of HFD fed Il22ra1fl/fl;Albumin-cre mice.

S4A – B represent 2 independent experiments. S4A – B are generated from at least 3 independent experiments. S4A is generated from N = 5 cre- and 6 cre+ mice. S4B is generated from 7 cre- and 10 cre+ mice. S4C is generated from 3 independent experiments and N = 6 cre- and 4 cre+ mice. 2-way ANOVA with Sidak multiple comparison in S4A, C; Mann-Whitney test, two-tailed in S4B. Data are presented as mean ± SEM in all graphs.

Gaudino et al Supplementary Figure 5

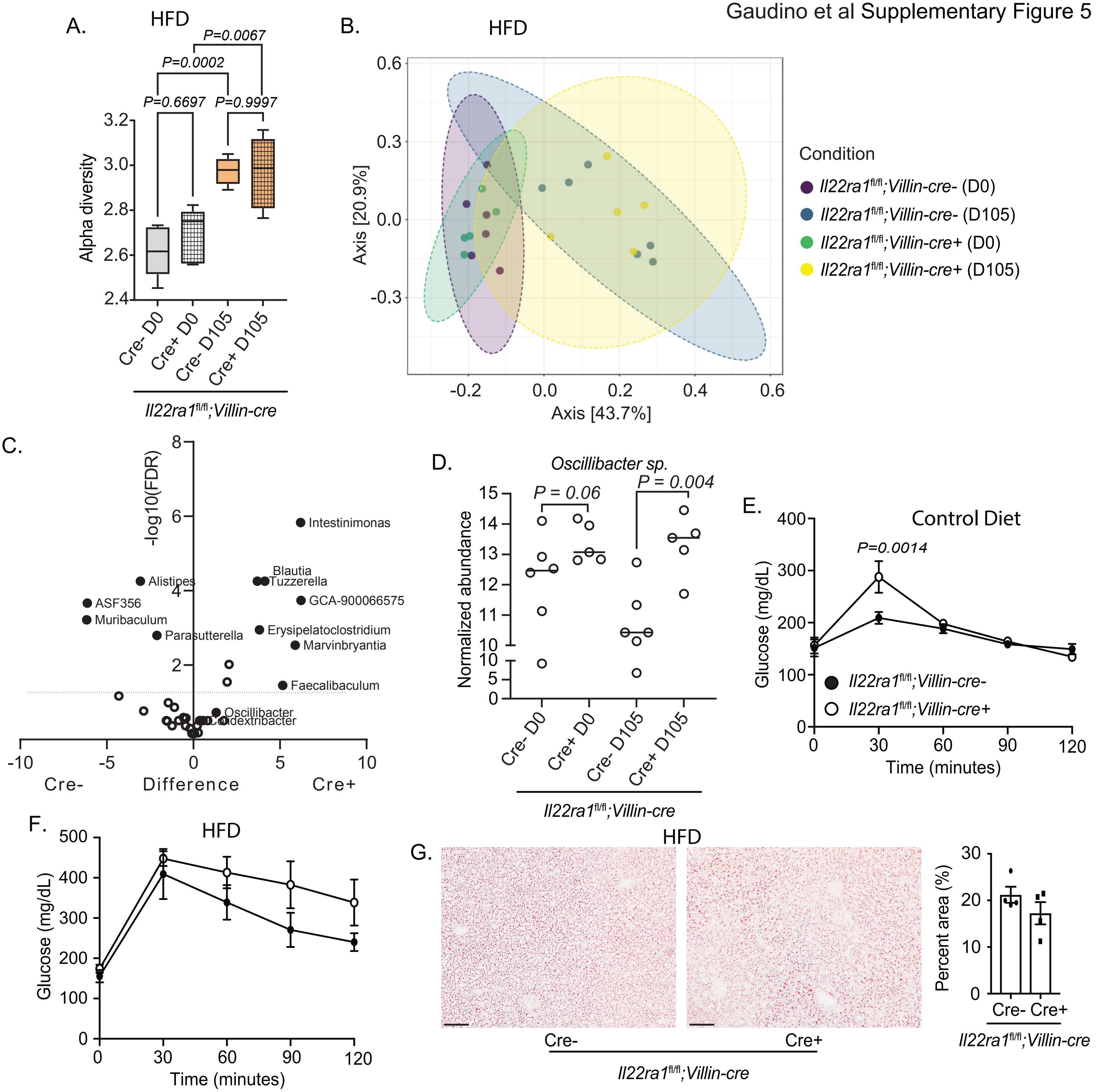

Supplementary Fig. 5. Microbiota analysis. (A) Genus-level alpha diversity analysis of 16S rRNA sequencing data derived from fecal DNA of paired Il22ra1fl/fl;Villin-cre mice before (day 0) and after (day 105) HFD. (B) Genus-level beta diversity analysis of 16S rRNA sequencing analysis derived from fecal DNA of paired Il22ra1fl/fl;Villin-cre mice. (C) Volcano plot of fecal 16S data displaying differential expression of bacterial genera from Il22ra1fl/fl;Villin-cre mice before long-term HFD treatment. (D) Normalized abundance of Oscillibacter before (day 0) and after (day 105) HFD. (E) GTT of germ-free mice that underwent a cecal microbiota transfer with contents either from long-term HFD fed Il22ra1fl/fl;Villin-cre-/+ mice. (F) GTT from long-term HFD fed Il22ra1fl/fl;Villin-cre mice under cohousing conditions. (G) ORO staining and quantification of HFD and antibiotics-treated Il22ra1fl/fl;Villin-cre mice.

S5A-D are generated from 2 independent experiments and N = 6 cre- and 5 cre+ mice. S5B utilizes Bray-Curtis analysis. S5E is generated from N = 4 cre- and 4 cre+ mice and 1 independent experiment. S5F represents N = 3 cre- and 3 cre+ mice and 3 independent experiments. S5G represents N = 4 cre- and 4 cre+ mice and 2 independent experiments. 2-way ANOVA in S6E-F; Mann-Whitney test, two-tailed in S5G; Mann-Whitney test, one-sided in S5D; Unpaired t-test in S5C. Scale bar = 50 μm. Error bars represent mean ± SEM.

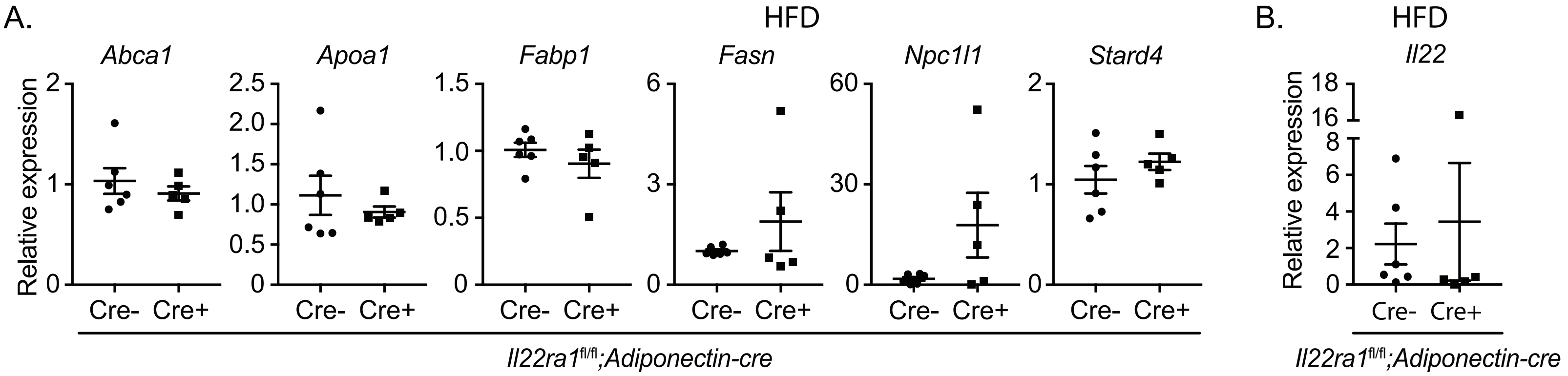

Supplementary Fig. 6. WAT-specific IL-22RA1 signaling. (A) RT-PCR analysis of metabolic genes from epididymal WAT of I22ra1fl/fl;Adiponectin-cre-/+ mice fed a long-term HFD. (B) RT-PCR analysis of Il22 transcript levels from WAT of I22ra1fl/fl;Adiponectin-cre-/+ mice fed a long-term HFD.

S6A – B represent N = 6 cre- and 5 cre+ mice and 2 independent experiments. Mann-Whitney test, two-tailed in S6A, C. Data are presented as mean ± SEM in all graphs.

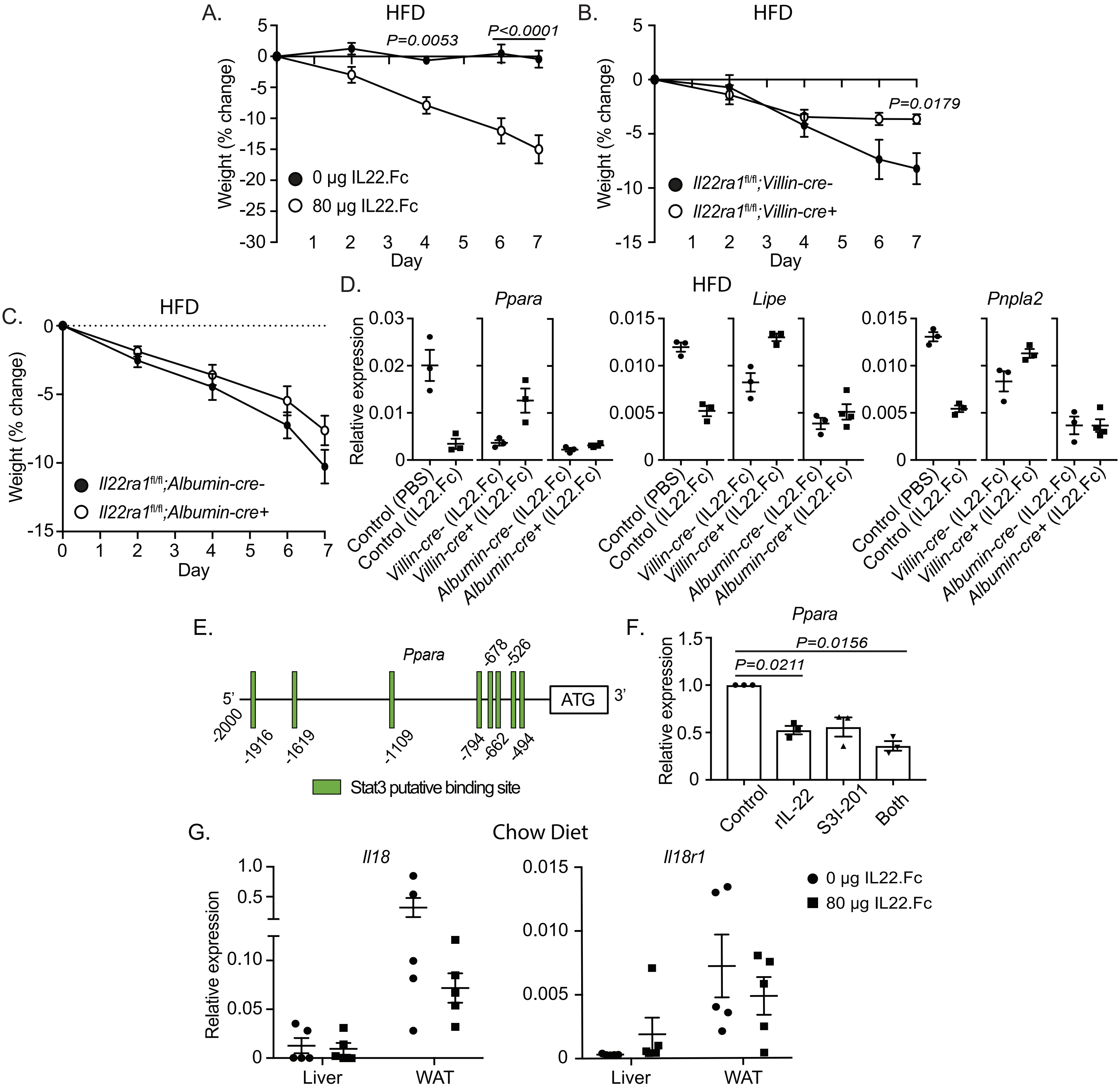

Supplementary Fig. 7. Regulation of intestinal lipid metabolism by IL-22. (A) Body weight of control (Lgr5-EG-FP-IRES-CreERT2) mice fed a HFD for 6 weeks and treated with 0 or 80 µg IL22.Fc every other day starting week 5. (B-C) Body weight of Il22ra1fl/fl;Villin-cre mice (B) or Il22ra1fl/fl;Albumin-cre mice (C) fed a HFD for 6 weeks and treated with 80 µg IL22.Fc every other day starting week 5. (D) RT-PCR of lipid metabolism genes from ileum of mice in S7A-C. (E) Putative murine Stat3 binding sites for Ppara. (F) RT-PCR of Ppara from wildtype small intestinal organoids treated with control media, 10 ng rIL-22, 100 µM S3I-201, or rIL-22 and S3I-201. (G) RT-PCR of Il18 (left) and Il18r1 (right) from liver and epididymal WAT from C57BL/6 mice injected with or without 80 µg IL22.Fc.

S7A represents 2 independent experiments and N = 3 mice in each group. S7B represents N = 3 cre- and 3 cre+ mice. S7C represents N = 3 cre- and 4 cre+ mice. S7D represents N = 3 Control, Villin-cre-/+, and Albumin-cre- mice and 4 Albumin-cre+ mice. S7F represents N = 3 mice. S7B – D and S7F were derived from 1 experiment. S7G represents N = 5 mice in each group and 2 independent experiments. Mann-Whitney test, two-tailed in S7D; 2-way ANOVA with Sidak multiple comparison in S7A-C, G; One-way ANOVA with Tukey multiple comparison in S7F. Data is presented as ±SEM in all graphs.

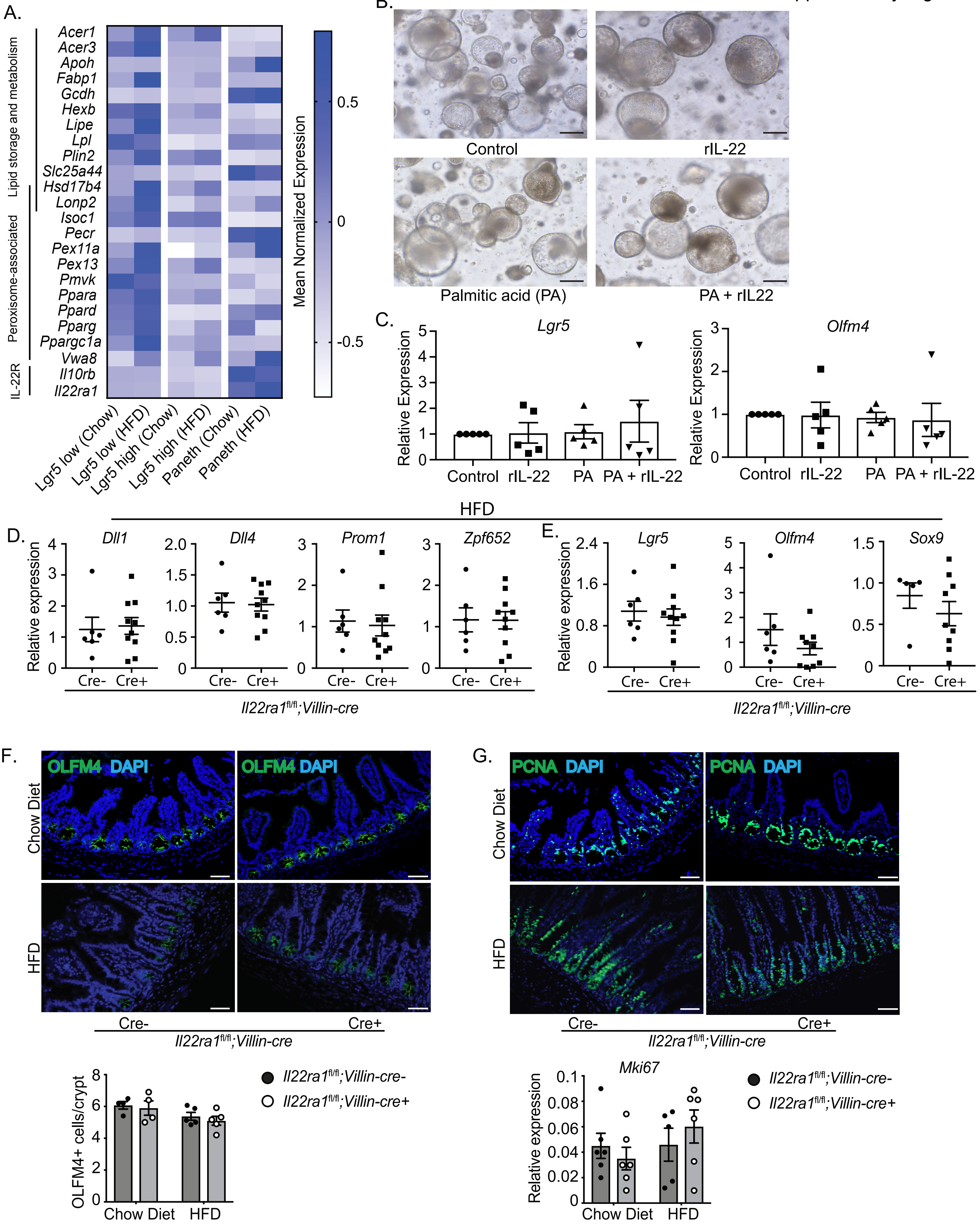
A.
Lipid storage and metabolism
Peroxisome-associated
IL-22R
Acer1
Acer3
Apoh
Fabp1
Gcdh
Hexb
Lipe
Lpl
Plin2
Slc25a44
Hsd17b4
Lonp2
Isoc1
Pecr
Pex11a
Pex13
Pmvk
Ppara
Ppard
Pparg
Ppargc1a
Vwa8
Il10rb
Il22ra1
Lgr5 low (Chow)
Lgr5 low (HFD)
Lgr5 high (Chow)
Lgr5 high (HFD)
Paneth (Chow)
Paneth (HFD)
Mean Normalized Expression
0.5
0
-0.5
B.
Control
rIL-22
Palmitic acid (PA)
PA + rIL22
C.
Lgr5
Olfm4
Relative Expression
Control
rIL-22
PA
PA + rIL-22
HFD
D.
Dll1
Dll4
Prom1
Zpf652
Relative expression
Cre-
Cre+
Il22ra1fl/fl;Villin-cre
E.
Lgr5
Olfm4
Sox9
Relative expression
Cre-
Cre+
Il22ra1fl/fl;Villin-cre
F.
OLFM4 DAPI
Chow Diet
HFD
Cre-
Cre+
Il22ra1fl/fl;Villin-cre
OLFM4+ cells/crypt
Chow Diet
HFD
Il22ra1fl/fl;Villin-cre-
Il22ra1fl/fl;Villin-cre+
G.
PCNA DAPI
Chow Diet
HFD
Cre-
Cre+
Il22ra1fl/fl;Villin-cre
Mki67
Relative expression
Chow Diet
HFD
Il22ra1fl/fl;Villin-cre-
Il22ra1fl/fl;Villin-cre+

Supplementary Fig. 8. IL-22RA1 signaling does not regulate ISC gene expression during fatty acid exposure. (A) Analysis of publicly available (GSE67324) RNA-seq data from sorted small intestinal Lgr5-GFP low (progenitor), Lgr5-GFP high (ISC), and Paneth cells from Lgr5-EGFP-IRES-CreERT2 mice fed a long-term (9 - 14 months) control diet or HFD. The expression of genes associated with lipid storage and metabolism, peroxisome function, and IL-22R from sorted cells was analyzed. (B) Representative images of wildtype ileal organoids treated with pure media, 10 μg rIL-22, 30 μM palmitic acid (PA), or 30 μM PA and 10 μg rIL-22 taken day 6 post plating. (C) RT-PCR analysis of Lgr5 (left) and Olfm4 (right) expression from day 6 organoids cultured in S8B. Data depict the fold change of the treated groups compared to the control group. (D) RT-PCR analysis of progenitor cell-associated genes from ileal tissues of HFD fed Il22ra1fl/fl;Villin-cre mice. (E) RT-PCR analysis of ISC-associated genes from ileal tissues of HFD fed Il22ra1-fl/fl;Villin-cre mice. F) Representative immunofluorescence images (left) and quantification (right) of OLFM4 stained ileal tissues from HFD fed Il22ra1fl/fl;Villin-cre mice. (G) Representative immunofluorescence images of PCNA stained ileal tissues from HFD fed Il22ra1fl/fl;Villin-cre mice (left) and RT-PCR analysis of Mki67 (right).

S8A is generated from RNA isolated by sorting 200,000 Lgr5-GFP high ISCs, Lgr5-GFP low progenitor cells, and Paneth cells from control diet or HFD fed Lgr5-EGFP-IRES-CreERT2 mice (Beyaz et al., 2016). N = 2 mice per group. S8B-C is generated from N = 5 mice and 2 independent experiments. S8D-E are generated from N = 6 cre- and 9 – 10 cre+ mice as well as 4 independent experiments. S8F represents N = 4 cre- and 4 cre+ mice (chow diet) and 5 cre- and 5 cre+ mice (HFD) as well as 2 (chow) and 3 (HFD) independent experiments. S8G (top) is representative of N = 4 cre- and 4 cre+ mice (chow diet) and 4 cre- and 7 cre+ mice (HFD) as well as 2 (chow) and 4 (HFD) independent experiments. S8G (bottom) is generated from N = 6 cre- and 6 cre+ mice (chow diet) and 5 cre- and 6 cre+ mice (HFD) as well as 3 independent experiments. Mann-Whitney test, two-tailed in S8D; One-way ANOVA with Tukey multiple comparison in S8C; 2-way ANOVA with Sidak multiple comparisons in S8F-G (bottom). Scale bar (S8B) = 100 μm; Scale bar (S8D-E) = 50 μm. Data is presented as ±SEM in all graphs.

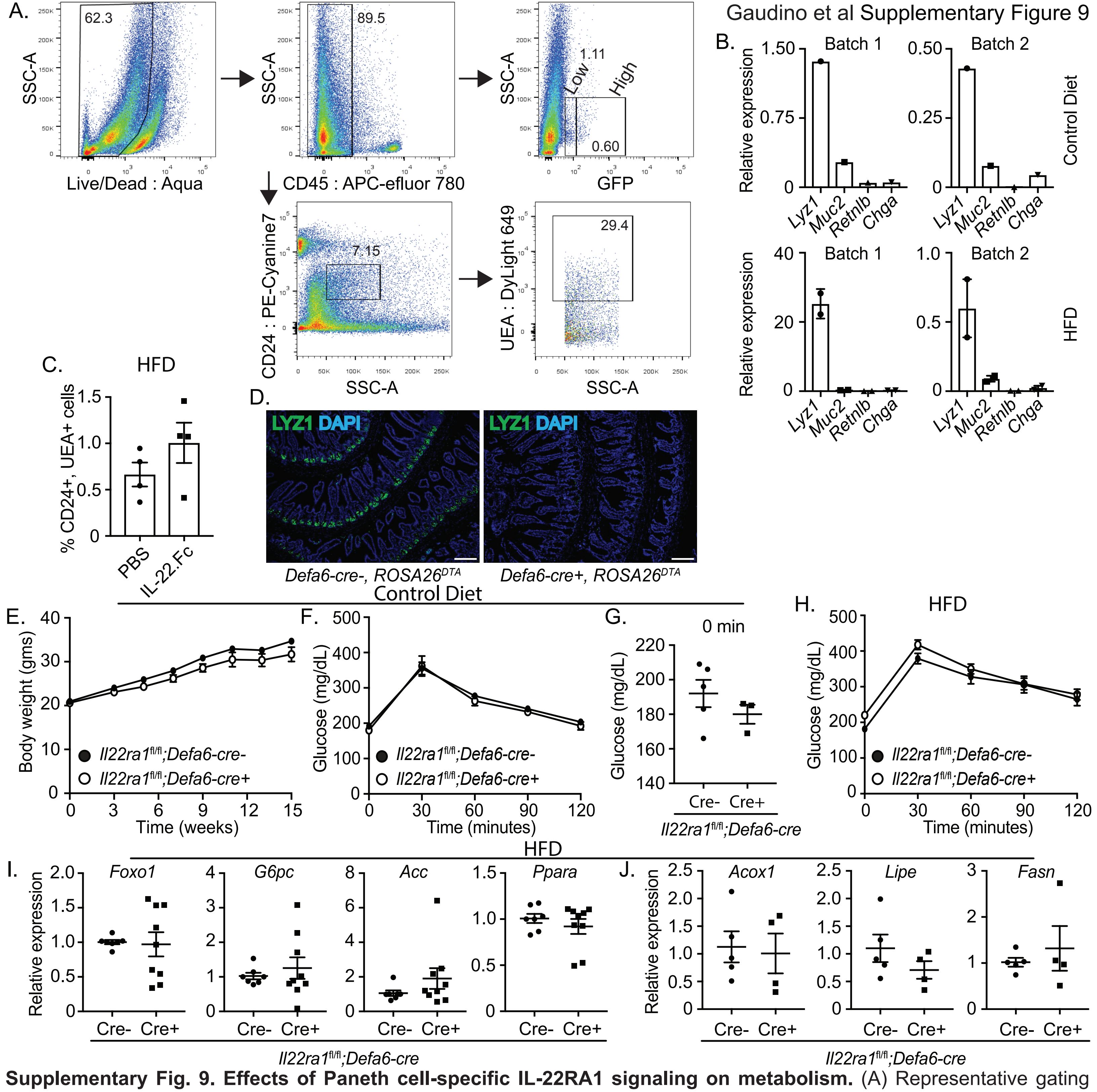

Supplementary Fig. 9. Effects of Paneth cell-specific IL-22RA1 signaling on metabolism. (A) Representative gating strategy for sorting Lgr5-GFP high (stem), Lgr5-GFP low (progenitor), and Paneth (CD24+, UEA+) cells. Small intestinal single cells were isolated from non-tamoxifen treated Il17rafl/fl;Lgr5-EGFP-creERT2+ or Il22ra1fl/fl;Lgr5-EGFP-creERT2+ mice for flow cytometry sorting and treated with 0 or 80 ug IL-22.Fc. (B) RT-PCR validation of sorting enrichment for Paneth cells (Lyz1) after control diet and HFD. (C) Percent of Paneth cells (CD24+, UEA+) from total sorted cells from mice fed a HFD. (D) Confirmation of Paneth cell knockout mice. (E) Body weight of Il22ra1fl/fl;Defa6-cre mice fed a control diet over a 15 week period. (F) GTT of Il22ra1fl/fl;Defa6-cre mice fed a control diet for 16 weeks. (G) Fasting blood glucose levels of Il22ra1fl/fl;Defa6-cre mice fed a control diet for 16 weeks. (H) GTT of Il22ra1fl/fl;Defa6-cre mice fed a HFD for 16 weeks. (I-J) RT-PCR of liver (I) and WAT (J) metabolic genes from HFD fed I22ra1fl/fl;Defa6-cre mice.

For S9B, 2 independent trials are portrayed (right and left batches) and N = 1 (control diet, both trials) and 2 (HFD, both trials). S9C represents N = 4 mice in each group and 2 independent experiments. For S9E – G, N = 5 cre- and 3 cre+ mice. S9H – I represent 4 independent experiments. S9H represents N = 11 cre- and 11 cre+ mice. S9I represents N = 7 cre- and 9 cre+ mice. S9J represents N = 5 cre- and 4 cre+ mice and 2 independent experiments. Mann-Whitney test, two-tailed in S9C, G, I-J; 2-way ANOVA with Sidak multiple comparison in S9E-F, H. Error bars represent ±SD in S9B and ±SEM in all other graphs. Scale bar = 100 μm.

Diet	Nutrition	Percent (%)
High Fat Diet (S3282)	Protein	20.5
	Fat	36.0
	Fiber	0.0
	Ash	3.5
	Moisture	< 10.0
	Carbohydrate	35.7
Control Diet (F4031)	Protein	20.5
	Fat	7.2
	Fiber	0.0
	Ash	3.5
	Moisture	< 10.0
	Carbohydrate	61.6

Supplementary Table 1. (A) Nutritional composition of Bio-Serve high fat diet (S3282) and control diet (F4031).

Target	Forward (5'-3')	Reverse (5'-3')	Vendor
Abca1	AAAACCGCAGACATCCTTCAG	CATACCGAAACTCGTTCACCC	IDT
Acc	AGCAGATCCGCAGCTTG	ACCTCTGCTCGCTGAGTGC	IDT
Acox1	CGCACATCTTGGATGGTAGT	GGCTTCGAGTGAGGAAGTTATAG	IDT
Apoa1	GGGACGTATGGCAGCAAGAT	CCAAGGAGGAGGATTCAAACTG	IDT
Dll1	GCAGGACCTTCTTTCGCGTAT	AAGGGGAATCGGATGGGGTT	IDT
Dll4	TTCCAGGCAACCTTCTCCGA	ACTGCCGCTATTCTTGTCCC	IDT
Emr1	CCCCAGTGTCCTTACAGAGTG	GTGCCCAGAGTGGATGTCT	IDT
Fabp1	ATGAACTTCTCCGGCAAGTACC	CTGACACCCCCTTGATGTCC	IDT
Fasn	TTCCAAGACGAAAATGATGC	AATTGTGGGATCAGGAGAGC	IDT
Foxo1	GCAGCCAGGCATCTCATAA	CCTACCATAGCCATTGCAGC	IDT
Gapdh	TGACCTCAACTACATGGTCTACA	CTTCCCATTCTCGGCCTTG	IDT
Il18	GTGAACCCCAGACCAGACTG	CCTGGAACACGTTTCTGAAAGA	IDT
Il18r1	TCACCGATCACAAATTCATGTGG	TGGTGGCTGTTTCATTCCTGT	IDT
Il22	TCCGAGGAGTCAGTGCTAAA	AGAACGTCTTCCAGGGTGAA	IDT
Lipe	CATCAACCACTGTGAGGGTAAG	AAGGGAGGTGAGATGGTAACT	IDT
Mmp12	CTGCCACTGTCCCAGGAAG	GGGAGAGTTTTCCAGTCATGG	IDT
Npc1l1	CGGAACTCACAGGACTTTACAG	TGCTGGTAGAACACATTGGAG	IDT
Olfm4	CAGCCACTTTCCAATTTCACTG	GCTGGACATACTCCTTCACCTTA	IDT
Pnpla2	CTTTGTGGCACAGACCTCTAA	GGGAGTAGTCGATGGAGAAGATA	IDT
Ppara	TTTCGGCGAACTATTCGGCTG	GGCATTTGTTCCGGTTCTTCTT	IDT
Pparg	AGGCGAGGGCGATCTTGACAG	AATTCGGATGGCCACCTCTTTG	IDT
Prom1	CTCCCATCAGTGGATAGAGAACT	ATACCCCCTTTTGACGAGGCT	IDT
Reg3b	ATGCGTCCTACTGCTATGCC	GTGTCCTCCAGGCCTCTT	IDT
Stard4	AGCCCGTGGTCACAGATTG	GCAACTCGCCACTCATCTTC	IDT
Zpf652	GGAGCTGGTTGAACCCTGTG	AGGGCTTCCAGACTCCCTTTT	IDT
Mki67 (Mm_Mki67_1_SG)	N/A	N/A	Qiagen (Cat#: QT00247667)
Hprt (Mm00446968_m1)	N/A	N/A	Applied Biosystems (Cat#: 4331182)
Lgr5 (Mm00438890_m1)	N/A	N/A	Applied Biosystems (Cat#: 4331182)
Lyz1 (Mm.PT.58.7374112)	N/A	N/A	IDT
Mmp7 (Mm.PT.58.29138073)	N/A	N/A	IDT
Reg3g (MM0041127_m1Reg3g	N/A	N/A	IDT
Il22ra1 (Mm.PT.58.42129001)	N/A	N/A	IDT
G6PC (Rxn Ready Primer Pool)	N/A	N/A	IDT

Supplementary Table 2. Primer list for RT-PCR analysis.
