# Supplementary material for: Intestinal IL-22RA1 signaling regulates intrinsic and systemic lipid and glucose metabolism to alleviate obesity-associated disorders
Source: Nat Commun. 2024 Feb 21;15:1597. doi: 10.1038/s41467-024-45568-6 (PMC10881576; doi:10.1038/s41467-024-45568-6)
Supplement: Supplementary file 2 — Reporting Summary [file 41467_2024_45568_MOESM2_ESM.pdf]

nature portfolio

Corresponding author(s): Pawan Kumar

Last updated by author(s): Dec 15, 2023



# Reporting Summary

Nature Portfolio wishes to improve the reproducibility of the work that we publish. This form provides structure for consistency and transparency in reporting. For further information on Nature Portfolio policies, see our Editorial Policies and the Editorial Policy Checklist.

## Statistics

For all statistical analyses, confirm that the following items are present in the figure legend, table legend, main text, or Methods section.

| n/a | Confirmed | |
|---|---|---|
| ☐ | ☒ | The exact sample size (*n*) for each experimental group/condition, given as a discrete number and unit of measurement |
| ☐ | ☒ | A statement on whether measurements were taken from distinct samples or whether the same sample was measured repeatedly |
| ☐ | ☒ | The statistical test(s) used AND whether they are one- or two-sided<br>*Only common tests should be described solely by name; describe more complex techniques in the Methods section.* |
| ☐ | ☒ | A description of all covariates tested |
| ☐ | ☒ | A description of any assumptions or corrections, such as tests of normality and adjustment for multiple comparisons |
| ☐ | ☒ | A full description of the statistical parameters including central tendency (e.g. means) or other basic estimates (e.g. regression coefficient) AND variation (e.g. standard deviation) or associated estimates of uncertainty (e.g. confidence intervals) |
| ☐ | ☒ | For null hypothesis testing, the test statistic (e.g. *F*, *t*, *r*) with confidence intervals, effect sizes, degrees of freedom and *P* value noted<br>*Give P values as exact values whenever suitable.* |
| ☒ | ☐ | For Bayesian analysis, information on the choice of priors and Markov chain Monte Carlo settings |
| ☒ | ☐ | For hierarchical and complex designs, identification of the appropriate level for tests and full reporting of outcomes |
| ☒ | ☐ | Estimates of effect sizes (e.g. Cohen's *d*, Pearson's *r*), indicating how they were calculated |

*Our web collection on statistics for biologists contains articles on many of the points above.*

## Software and code

Policy information about availability of computer code

| | |
|---|---|
| Data collection | 16S rRNA microbial analysis: Analysis utilizing PCR amplification of the V4 region of 16S rRNA followed by sequencing on an Illumina MiSeq. |
| Data analysis | 16S rRNA microbial analysis: Taxonomy assignment was performed using the Silva Project's version 138.1 release and formatted for use with DADA2 (v1.21). The downstream and statistical analysis was carried out with Phyloseq (v1.34.0), quantile normalization and differential abundance using BRB-ArrayTools (https://brb.nci.nih.gov/BRBArrayTools/) and alpha and beta diversity using MicrobiomeAnalyst61.<br><br>Promoter binding site prediction: JASPAR (https://jaspar.genereg.net/) and NCBI were used to identify predicted binding sites.<br><br>Statistical analysis was performed using GraphPad Prism Version 7.05.237. |

For manuscripts utilizing custom algorithms or software that are central to the research but not yet described in published literature, software must be made available to editors and reviewers. We strongly encourage code deposition in a community repository (e.g. GitHub). See the Nature Portfolio guidelines for submitting code & software for further information.

## Data

Policy information about availability of data

All manuscripts must include a data availability statement. This statement should provide the following information, where applicable:

- Accession codes, unique identifiers, or web links for publicly available datasets
- A description of any restrictions on data availability
- For clinical datasets or third party data, please ensure that the statement adheres to our policy

16S rRNA microbial sequencing was deposited in the NCBI BioProject database (ID: PRJNA944165). Small intestinal RNA-sequencing data previously deposited in the GEO database (accession number: GSE67324) was analyzed.

## Human research participants

Policy information about studies involving human research participants and Sex and Gender in Research.

| | |
|---|---|
| Reporting on sex and gender | No human participants were involved in this research |
| Population characteristics | No human participants were involved in this research |
| Recruitment | No human participants were involved in this research |
| Ethics oversight | No human participants were involved in this research |

Note that full information on the approval of the study protocol must also be provided in the manuscript.

# Field-specific reporting

Please select the one below that is the best fit for your research. If you are not sure, read the appropriate sections before making your selection.

☒ Life sciences ☐ Behavioural & social sciences ☐ Ecological, evolutionary & environmental sciences

For a reference copy of the document with all sections, see nature.com/documents/nr-reporting-summary-flat.pdf

# Life sciences study design

All studies must disclose on these points even when the disclosure is negative.

| | |
|---|---|
| Sample size | Sample size was not predetermined with statistical analyses. Approximate sample size was determined based on our previous experience utilizing Il22ra1 tissue-specific knockout and control mice for in vivo and organoid studies. A sample size of at least n = 3 was used for all experiments. |
| Data exclusions | Data points were only excluded if they were statistical outliers as determined by using GrapPad Prism (ROUT analysis, Q = 1%). |
| Replication | Experiments were performed ideally at least two times to confirm reproducibility. Similar results were obtained between experiments. In cases where experiments were performed once, this was due to limited resources to perform an additional round of experimentation. |
| Randomization | Mice were separated into groups based on their genotype status. Once genotypes were determined, mice within each group were randomly assigned for different experimental protocols. Wildtype mice were randomly assigned for organoid culture experiments. |
| Blinding | Researchers were blinded during data acquisition and analysis whenever possible including for GTTs, tissue staining and quantification, gas chromatography, and cell counting. |

# Reporting for specific materials, systems and methods

We require information from authors about some types of materials, experimental systems and methods used in many studies. Here, indicate whether each material, system or method listed is relevant to your study. If you are not sure if a list item applies to your research, read the appropriate section before selecting a response.

## Materials & experimental systems

| n/a | Involved in the study |
| --- | --- |
| ☐ | ☒ Antibodies |
| ☐ | ☒ Eukaryotic cell lines |
| ☒ | ☐ Palaeontology and archaeology |
| ☐ | ☒ Animals and other organisms |
| ☒ | ☐ Clinical data |
| ☒ | ☐ Dual use research of concern |

## Methods

| n/a | Involved in the study |
| --- | --- |
| ☒ | ☐ ChIP-seq |
| ☐ | ☒ Flow cytometry |
| ☒ | ☐ MRI-based neuroimaging |

# Antibodies

| | |
| --- | --- |
| Antibodies used | anti-CD45:APC-eFluor780, Invitrogen, Ref: 47-0451-82 was used for flow cytometry, dilution = 1:200<br>anti-CD24:PE-Cyanine7, Invitrogen, Ref: 25-0242-80 was used for flow cytometry, dilution = 1:200<br>anti-Lyz1-FITC, Dako , Ref: F0372 was used for immunofluorescence, dilution = 1:200<br>anti-OLFM4, Cell Signaling Technology, Ref: 39141S was used for immunofluorescence, dilution = 1:200<br>anti-PCNA, Santa Cruz Biotecnology, Ref: Sc-56 was used for immunofluorescence, dilution = 1:200<br>anti-F4/80, Miltenyi Biotec, Ref: 130-117-509 was used for immunofluorescence, dilution = 1:200<br>anti-MMP12, Abcam, Ref: ab231109 was used for immunofluorescence, dilution = 1:50<br>anti-rabbit IgG Fab2 Alexa Fluor® 488, Cell Signaling Technology, Ref: 4412 was used for immunofluorescence, dilution = 1:200 |
| Validation | All antibodies were previously validated by their manufacturers. Invitrogen antibodies were validated for flow cytometry with species specificity for mice. anti-OLFM4, anti-PCNA, anti-F4/80, and anti-rabbit IgG Fab2 Alexa Fluor® 488 were all validated for immunufluorecence staining with reactivity to murine tissues. We previously validated that anti-Lyz1-Fitc stains murine intestinal tissues for immunofluorescence analysis (Gaudino et al, Mucosal Immunol, https://doi.org/10.1038/s41385-020-00348-5). anti-MMP12 was validated for murine western blot analysis by its vendor. Our lab tested all antibodies for efficacy and appropriate dilution factors (listed above and in manuscript text). |

# Eukaryotic cell lines

Policy information about cell lines and Sex and Gender in Research

| | |
| --- | --- |
| Cell line source(s) | HepG2, ATCC |
| Authentication | STR profiling authentication was performed by the vendor. No additional authentication was performed. |
| Mycoplasma contamination | Cell lines tested negative for contamination |
| Commonly misidentified lines<br>(See ICLAC register) | No commonly misidentified cell lines were used in this study. |

# Animals and other research organisms

Policy information about studies involving animals; ARRIVE guidelines recommended for reporting animal research, and Sex and Gender in Research

| | |
| --- | --- |
| Laboratory animals | All mouse (Mus musculus) strains used were generated on a C57BL6 background. The following murine strains were used: Il22-/-, Il22ra1fl/fl;Albumin-cre, Il22ra1fl/fl;Villin-cre, Il22ra1fl/fl;Adiponectin-cre, Lgr5-EGFP-IRES-CreERT2, Il22ra1fl/fl;Defa6-cre, and Defa6-cre+; ROSA26DTA. All mice were at least 6 weeks old when used for experiments (including placement on chow, control, or high fat diets). All mice were housed under a 12/12 light/dark cycle, 64 – 79°F, and 30 – 70% humidity. Animal studies were conducted with the approval and under all relevant ethical regulations of Stony Brook University's Institutional Animal Care and Use Committee. |
| Wild animals | Study did not involve wild animals. |
| Reporting on sex | Male mice were solely used for control diet and high fat diet experiments to control for previously reported sex-dependent metabolic differences. The use of male/female mice is indicated in the manuscript. |
| Field-collected samples | Study did not involve field-collected samples. |
| Ethics oversight | Animal studies were conducted with the approval and under all relevant ethical regulations of Stony Brook University's Institutional Animal Care and Use Committee. |

Note that full information on the approval of the study protocol must also be provided in the manuscript.

## Flow Cytometry

### Plots

Confirm that:

☒ The axis labels state the marker and fluorochrome used (e.g. CD4-FITC).

☒ The axis scales are clearly visible. Include numbers along axes only for bottom left plot of group (a 'group' is an analysis of identical markers).

☒ All plots are contour plots with outliers or pseudocolor plots.

☒ A numerical value for number of cells or percentage (with statistics) is provided.

### Methodology

| | |
|---|---|
| Sample preparation | Small intestinal murine crypts were isolated from 10 cm ileal tissue. Crypts were dissociated into single cells using TrypLe/ DNase digestion followed by thorough separation by pipetting. Cells were appropriately stained for 30 minutes. Cells were filtered directly before acquisition. |
| Instrument | BD FACS ARIA Cell Sorter |
| Software | BD FACS DIVA 9.0.1 software was used |
| Cell population abundance | Total abundance of Lgr5+ intestinal stem cells and Paneth cells is relatively low compared to the overall number of cells sorted. However, this is expected since there are only about 4-6 intestnal stem cells and 5-12 Paneth cells in a healthy crypt. |
| Gating strategy | Gating strategy was based on prior literature. |

☒ Tick this box to confirm that a figure exemplifying the gating strategy is provided in the Supplementary Information.
